# X- and Y-chromosome specific variants of the amelogenin gene allow sex determination in sheep (*Ovis aries*) and European red deer (*Cervus elaphus*)

**DOI:** 10.1186/1471-2156-6-16

**Published:** 2005-03-16

**Authors:** I Pfeiffer, B Brenig

**Affiliations:** 1 Department of Molecular Biology, Institute of Veterinary Medicine, Goettingen, Germany

## Abstract

**Background:**

Simple and precise methods for sex determination in animals are a pre-requisite for a number of applications in animal production and forensics. However, some of the existing methods depend only on the detection of Y-chromosome specific sequences. Therefore, the abscence of a signal does not necessarily mean that the sample is of female origin, because experimental errors can also lead to negative results. Thus, the detection of Y- and X-chromosome specific sequences is advantageous.

**Results:**

A novel method for sex identification in mammals (sheep, *Ovis aries *and European red deer, *Cervus elaphus*) is described, using a polymerase chain reaction (PCR) and sequencing of a part of the amelogenin gene. A partial sequence of the amelogenin gene of sheep and red deer was obtained, which exists on both X and Y chromosomes with a deletion region on the Y chromosome. With a specific pair of primers a DNA fragment of different length between the male and female mammal was amplified.

**Conclusion:**

PCR amplification using the amelogenin gene primers is useful in sex identification of samples from sheep and red deer and can be applied to DNA analysis of micro samples with small amounts of DNA such as hair roots as well as bones or embryo biopsies.

## Background

Sex identification using genomic DNA extracted from meat, blood, hair or embryo biopsies is sometimes an important analytical tool in forensic science or in routine genotyping. In most mammals, the male is identified by amplifying the SRY gene (sex-determining region Y) which is a Y chromosome-specific sequence. When the results are negative from amplification of only the SRY gene, it cannot be assumed that the individual is female or that there was a mistake in the experimental process. A gene present in both, males and females, should be amplified in the same tube as a positive control. In sex identification of ursides by PCR, primers that amplify the SRY gene together with the mitochondrial DNA control region or the ZFX/Y region have been used [1,2]. Furthermore, recent reports have described the use of low-stringency PCR or the detection of X/Y specific restriction fragment polymorphisms (RFLP) in sheep and goats [3,4]. However, RFLP analysis requires an additional reaction step which might increase the risk of contamination and misdiagnosis. The amelogenin (AMEL) gene, which exists on both X and Y chromosomes, has been used to determine the sex in cattle [5] and humans [6]. The use of this gene has made the sex determination much less complicated, since only one pair of primers is required to amplify the different size fragments of the AMEL genes.

In this study we used the primers established in cattle to determine their suitability for other species, i.e. sheep and European red deer. The amplicons were isolated and sequenced, and showed a length polymorphism characteristic for the X and Y chromosome in both species.

## Results

**Figure 1:**
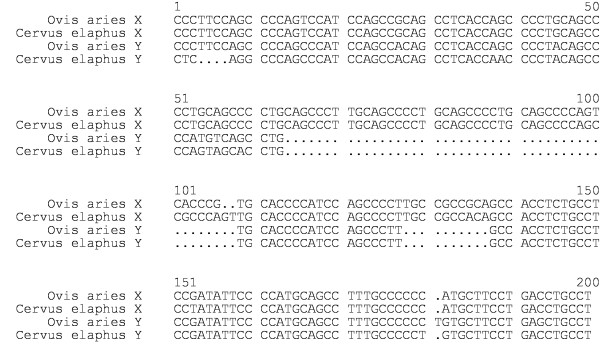
DNA sequence comparison of the X and Y amelogenin gene fragments of sheep and European red deer. (.) ins/del

The nucleotide sequences of the PCR products of the sheep and the red deer amelogenin gene are shown in Figure 1. Both male specific products harbour different basepair deletions (ins/del). Besides a few minor single nucleotide ins/dels, the most prominent ins/del is between positions 63 and 107 (45 bp) and positions 127 and 136 (10 bp) according to Fig. 1. This major ins/del leads to a shorter amplicon that can easily be detected in an agarose gel. The sheep and red deer male specific product was deposited with the EMBL/Genebank database under accession numbers AY453392 and AY453391, respectively. The comparison of the sheep and red deer with the cattle amelogenin gene DNA sequences showed a 96 % and 97 % homology for the AMELX gene and a 86 % and 90 % homology for the AMELY gene, respectively.

**Figure 2:**
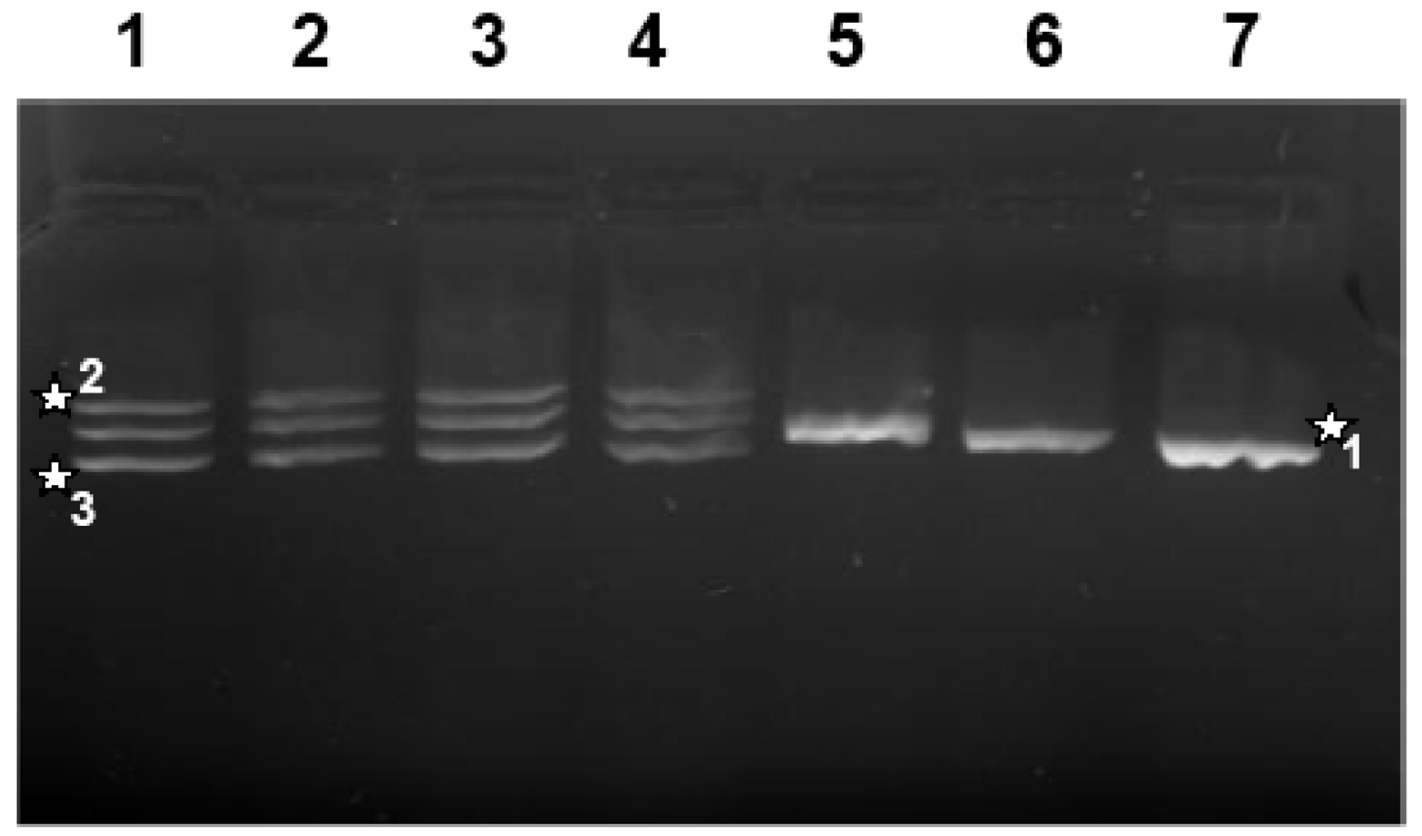
Detail of an ethidiume bromide stained agarose gel showing amplicons of the amelogenin gene fragments of sheep and European red deer. Lane 1–2 samples from male red deer, lane 3–4 samples from male sheep, lane 5 sample from female sheep and lane 6–7 samples from female red deer. The star 1 indicates the AMELX band, the star 2 indicates a non specific band in male sheep and male red deer and the star 3 indicates the AMELY band.

As expected, a single band for females (EMBL/Genebank accession number female sheep AY 452664 and female red deer AY 452665) and two bands for the males were detected when analysing different animal samples (Fig. 2). Although an additional non-specific band was observed in male sheep and red deer, there was no problem to determine the sex, based on the different banding patterns.

## Discussion

In this study we have established a simple and accurate method for determining the sex of sheep and red deer by using a pair of primers for the AMEL gene. The amelogenin gene encodes an important protein in the developing mammalian tooth enamel matrix [7,8] that has been conserved during the evolution of vertebrates. The bovine amelogenin gene [9] is located on the X and Y chromosomes, and differences in their length are observed between the X and Y specific gene. In other species analysed so far, e.g. mice [10], the gene is located only on the X chromosome. Our results show that the amelogenin gene of sheep and red deer is located on both sex chromosomes and that there are two diagnostic ins/del of 55 bp together within the Y-specific gene in the region amplified.

By using this method in combination with routine genotyping more information about a material under investigation can be obtained. In addition, the amplification of the AMEL gene can also be used as an internal control. Furthermore, the contamination with human DNA is sometimes a problem during the laboratory analysis. However, the amplification of the human AMEL gene with the same primers resulted in a different and clearly distinguishable banding pattern. The conserved status of the amelogenin gene among vertebrates indicates the possibility to use the test in other wild mammal species as well.

## Conclusion

In conclusion our findings show that the PCR assay based on the AMEL gene is reliable for sex identification in sheep and European red deer. The advantage of this assay is that neither additional control amplicons with a second locus-specific autosomal primer pair nor restriction endonuclease steps are necessary for sex determination and control of the PCR reaction.

## Methods

Samples were taken from sheep (*Ovis aries*) and European red deer (*Cervus elaphus*). DNA was extracted from different tissue samples using QIAamp^® ^Tissue Kit (QIAGEN GmbH, Hilden, Germany) according to the manufacturers' handbook. Isolated DNA was diluted in 50 μl HPLC-H_2_O and used for further analyses.

We used one set of primers (SE47, SE48) for amplifying the sheep and red deer amelogenin gene. The DNA sequences of the primers were 5'-cagccaaacctccctctgc-3' (SE47) and 5'-cccgcttggtcttgtctgttgc-3' (SE48) as described by [5].

Amplifications were performed in a final volume of 20 μl in 10 × PCR buffer (15 mM MgCl_2_, pH 8.3) and Q-solution (QIAGEN GmbH, Hilden, Germany), 100 μM for each dNTP, with 1 M Taq DNA Polymerase and 10 pmol of each primer. Four microlitres of the DNA-extract were added to the PCR mix. The amplification was carried out with initial denaturation at 95°C for 10 min, followed by 35 cycles of one denaturation step at 94°C for 50 sec, primer annealing at 56°C for 50 sec and primer extension at 72°C for 50 sec in a Hybaid Omnigene thermocycler (MWG Biotech, Ebersberg, Germany). A final extension step was not included. PCR-products were purified using the QIAEX II Gel Extraction Kit (QIAGEN GmbH, Hilden, Germany) according to the manufacturers' instructions. Sequencing was performed using ABI-Prism™ BigDye Terminator v3.1 Cycle Sequencing Kit (Applied Biosystems, Weiterstadt, Germany) in a 10 μl volume containing 2 μl purified PCR-product and 5 pmol of primer. Sequencing reactions underwent 27 cycles of 30 sec at 94°C, 30 sec at 55°C and 3 min at 60°C in a Techne Gene E Thermocycler (Burkhardtstorf, Germany). The dye terminators were removed by sephadex-G45 column purification (Millipore). Sequencing reactions were electrophoresed for 2 h on an ABI Prism^® ^3100 Genetic Analyzer (Applied Biosystems, Weiterstadt, Germany) according to the manufacturers' instructions.

## Authors' contributions

IP performed the DNA extractions, PCR analysis and the DNA sequencing.

BB was responsible for funding, supervision of the research project, manuscript writing and editing as well as scientific correspondence.
